# Severe Anaphylactic Reaction to Diclofenac during Intravenous Anesthesia for In Vitro Fertilization

**DOI:** 10.1155/2019/8583753

**Published:** 2019-12-03

**Authors:** Nebojsa Videnovic, Nebojsa Markovic, Jovan Mladenovic, Marija Jovic, Rasa Mladenovic, Ranko Zdravkovic

**Affiliations:** ^1^Department of Anesthesiology and Intensive Care, Medical Faculty, University of Pristina – Kosovska Mitrovica, Serbia; ^2^Department of Gynecology, Spebo Medical Special Hospital for Fertility Treatments, Leskovac, Serbia; ^3^Department of Surgery, Medical Faculty, University of Pristina – Kosovska Mitrovica, Serbia; ^4^Department of Anesthesiology, General Hospital, Leskovac, Serbia; ^5^Department of Dentistry, Medical Faculty, University of Pristina – Kosovska Mitrovica, Serbia; ^6^Department of Anesthesiology, Clinic for Cardiovascular Surgery, Institute of Cardiovascular Diseases of Vojvodina, Sremska Kamenica, Serbia

## Abstract

We present a case of a severe anaphylactic reaction to diclofenac, administered as an intravenous infusion in a 45-year-old patient, during intravenous anesthesia for in vitro fertilization. During the preoperative clinical examination and obtaining of anamnestic data, the patient denied symptoms of allergies to medicines and other substances. The dominant clinical manifestations of anaphylactic reaction were: hypotension, tachycardia, angioedema, bronchospasm, and delayed awakening following anesthesia. No visible changes in the appearance of the skin, such as erythema or urticaria have been observed. Proper clinical observation and adequate intraoperative monitoring led to rapid diagnosis and significantly reduced the time interval from the onset of anaphylaxis to the beginning of treatment and reanimation procedures.

## 1. Introduction

Nonsteroidal anti-inflammatory drugs (NSAIDs) are among the most widely used drugs worldwide, involving a variety of agents that possess different chemical structures. Most of these drugs exert three main types of effects: anti-inflammatory effects—modification of the inflammatory response, analgesic effect—relief of certain types of pain, and antipyretic effect—reducing of fever. Generally, all of these are related to the primary effect of NSAIDs to inhibit cyclooxygenase and thereby inhibit the production of prostaglandins and thromboxane, although certain aspects of the effect can be achieved via different mechanisms; some of the drugs also exert other effects than those related to the inflammatory process.

Arachidonic acid metabolism takes place via the cyclo-oxygenase and the lipo-oxygenase pathway. Both the pathways produce potent mediators of a multitude of immune-induced and inflammatory reactions. Any blockade of cyclo-oxygenase pathway (COP) shunts the metabolism towards the lipo-oxygenase pathway (LOP) and can potentially increase the side-effects of that pathway by augmented production and release of cysteinyl-leukotriene [1, 2].

Common side effects include: dyspepsia, diarrhea/constipation, nausea, and vomiting, and in some cases bleeding from the stomach and ulcer, rash, urticaria, photosensitivity reactions, acute renal failure, analgesic nephropathy (in chronic use), bone marrow, and liver diseases. Diclofenac sodium belongs to NSAIDs and has wide application in the world as a nonopioid analgesic. It is considered a relatively safe drug with a small number of reported severe anaphylactic reactions. We report a case of severe anaphylactic reaction to diclofenac, administered as an intravenous infusion, during intravenous anesthesia for in vitro fertilization (IVF).

## 2. Case Report

A 36-year-old female patient, was undergoing IVF treatment in our institution. After hormonal stimulation of the ovaries, follicular aspiration was initiated in order to obtain ovarian cells. Oocyte retrieval involves direct ultrasound guidance, i.e., a needle is passed through the top of the vagina to reach the follicles. The patient denied symptoms of allergies to medicines, foods, and other substances during a routine clinical examination by an anesthetist. Anesthetic protocol implied fasting for 4 hours prior to the procedure. The patient received low molecular weight heparin preoperatively. Cubital vein cannulation was performed to enable the administration of fluids and medicaments. Hydration was provided by continuous infusion of Ringer's lactate solution (10 mL/kg body weight). The procedure itself was performed in short-term intravenous anesthesia.

After positioning, the patient is linked to the mandatory standard monitoring for this type of intervention listed above. After recording monitoring parameters from preinduction stage, the patient was premedicated with 0.02 mg/kg intravenous midazolam. Anesthesia was induced with propofol 2 mg/kg and alfentanil 0.01 mg/kg. Additional propofol was administered to maintain BIS values within the target range (40–60).

In the incidence of apnoea after induction of anesthesia, the patient was mechanically ventilated through a face mask or a cuffed oropharyngeal airway with tidal volume of 8 mL/kg body weight. The inspiratory mixture of oxygen and medical air delivered the inspired oxygen concentration of 40% (FiO_2_ 0.4).

The standard monitoring included: BIS index, pulse oximetry (SaO_2_), Level of (partial pressure) of carbon dioxide released at end of expiration (EtCO_2_), Peak inspiratory pressure (Ppeak), Plateau Airway Pressure (Pplato), tidal volume (Vt), mean arterial blood pressure (ABP), and electrocardiography (EKG). Clinical parameters were measured by vital sign monitor (Covidien BISTM Complete 2 Channel Monitor, Medtronic, and Monitor Infinity Gamma XL, Dräger) and anesthesia machine (FabiusTiro Anesthesia Machine, Dräger).

After the introduction to anesthesia, the gynecologist initiated follicular aspiration. Monitoring parameters were within reference ranges (ABP-113/68 mmHg, SaO_2_-99%, heart rate-75/min, EtCO_2_-34 mmHg, ECG-in the normal range, Pplato-10 mbar and Ppeak-12 mbar).

Due to the presence of a large number of ovarian follicles (>30), we decided to use diclofenac sodium (75 mg) as an intravenous infusion in order to prolong the postoperative analgesia. After initiating the infusion of the solution with diclofenac-sodium (1-2 min), we recorded increase in the heart rate (>160/min), a drop in blood pressure (<70/40 mmHg), with frequent occurring premature supraventricular and ventricular contractions in the ECG.

Pulse oximetry revealed the reduction in SaO_2_ from 100% to 68% followed with the decrease in end-tidal carbon dioxide (ETCO_2_) values (33 mmHg at 19 mmHg). A pronounced secretion from oral and nasal cavity was observed. Also, there was a significant increase in the Ppeak (from 12 mbar to 24 mbar) and Pplato values (10–20 mbar) during manual ventilation of the patient via a face mask and an oropharyngeal tube. Auscultation of chest revealed bilateral ronchi. No visible changes in the appearance of the skin, such as erythema or urticaria were observed. The development of edema in the lips, periorbital area, eyelids, and face was observed for a short time interval. Infusion of diclofenac was stopped; 2 doses of 0.5 mg adrenaline were administrated intramuscularly. Crystalloid solutions (Ringer lactate and 0.9% NaCl), methylprednisolone, chlorpyramine, and aminophylline were administered intravenously. The patient was ventilated with 100% oxygen to initiate emergence from anesthesia. During manual ventilation, significant decrease in airway resistance (reduction of Ppeak and Pplato values) was noted. Once the vital signs were stabilized, sufficient spontaneous breathing was established. The awakening was delayed and the patient regained consciousness after 60 minutes. During the reanimation, blood for the evaluation of serum troponins, D-dimer, and tryptase was sampled. Test for tryptase level was repeated on several occasions. Its highest values were observed after two hours from the onset of anaphylaxis (31 ng/mL). After 9 hours, tryptase values returned to the reference range (<11 ng/mL). Troponins and D-dimer remained in the normal range. A subsequent discussion with the patient revealed that she had a pronounced skin irritation in the area of the lower abdomen and lower extremities after intramuscular application of diclofenac a few years before. This was disregarded by the doctor at the time. We did not obtain this data during the preoperative examination of the patient. After 12 h, the patient was discharged from the hospital, with referral for additional allergologic tests.

Fifteen days after leaving the hospital, allergy tests were performed on the patient. Hypersensitivity to diclofenac sodium with increased IgE antibody titre has been demonstrated. At the same time, hypersensitivity to other medications and anesthetics administered during aspiration of the ovarian follicles was excluded.

## 3. Discussion

Hypersensitivity to NSAIDs has a spectrum of manifestations from urticaria to severe anaphylaxis that can be life-threatening. These reactions are more frequent in patients with a known allergic reaction or autoimmune disorders who are to be considered a population at risk [2]. Diclofenac by itself was considered a safe drug and the experience in terms of patient-years is huge. The side effect is similar to other NSAIDs. However, multiple cases of severe anaphylactic reactions after the application of diclofenac have been reported, even with fatal outcome [3]. These case reports indicate the possibility of an anaphylactic reaction to occur regardless of the route of administration. It can occur after intramuscular, oral, rectal, or intravenous administration of diclofenac sodium [3–6].

The authors of this text found no publications in English reporting cases of severe anaphylactic reactions to intravenously administered diclofenac in patients undergoing anesthesia, where we are unable to monitor alteration in conscious state. The cardiovascular effects of the anaphylactic reaction during anesthesia are further potentiated by the influence of anesthetics (popofol, fentanyl, alfentanil, and remifentanil) on myocardial and peripheral vascular depression [7, 8]. The presence of bronchospasm and angioedema led to an increase in airway resistance thus causing a significant increase in Ppeak and Pplato values during artificial ventilation of the patient. The ventilation-perfusion mismatch caused a significant drop in SaO_2_ and EtCO_2_ values. Hypoperfusion and hypoxemia CNS are closely related to the delayed awakening of patients from anesthesia.

An anaphylactic reaction may be a trigger for the development of myocardial infarction or acute coronary syndrome, even in people with normal coronary blood flow. Continuous ECG monitoring did not reveal changes in the ST-segment (depression/elevation) and T waves. Troponins stayed within reference range. A routine use of low molecular weight heparin as an integral part of the IVF procedure makes the pulmonary embolism unlikely [9]. Tachycardia, severe hypotension, or shock state can lead to the decrease in effective circulatory volume (bleeding). However, this is immediately excluded by ultrasound monitoring. Although the absence of skin changes (urticaria, erythema, etc.) can lead to difficulties in differential diagnostics, rapid development of angioedema with cardiovascular changes is a step in the right direction, especially considering the occurrence, and development of anaphylactic reaction only after injection of the diclofenac-sodium infusion.

Careful clinical assessment and continuous monitoring are important so as to avoid further morbidity and mortality [6]. Rapid diagnoses significantly reduces the time interval from the onset of anaphylaxis to the beginning of treatment and reanimation procedures [10, 11].

This case exemplifies that a properly taken medical history and a clinical examination of the patient do not exclude the possibility of allergic reactions to diclofenac sodium or other drugs. The reason is the inaccurate allergy history to the drug. Such medical errors could endanger the lives of patients.

## 4. Conclusion

Severe anaphylactic reactions may also occur in medication that is considered relatively safe. The diagnosis of anaphylactic reactions with the development of shock during anesthesia was further complicated by the anesthetic itself and burdened with differential-diagnostic dilemmas. Failures in preoperative preparation for surgical intervention, in terms of getting acquainted with possible allergic reactions, significantly affect their frequency.
